# Histopathological and Behavioral Assessment of Toxin-Produced Cerebellar Lesion: A Potent Model for Cell Transplantation Studies in The Cerebellum

**Published:** 2014-10-04

**Authors:** Mohammad Amin Edalatmanesh, Haniyeh Nikfarjam, Marzieh Moghadas, Aliakbar Haddad-Mashadrizeh, Reza Robati, Mohammad Reza Hashemzadeh

**Affiliations:** 1 Department of Physiology, Science and Research Branch, Islamic Azad University, Fars, Iran; 2 Department of Biology, Faculty of Science, Ferdowsi University of Mashhad, Mashhad, Iran; 3 Department of Stem cell and Regenerative Biology, Eram Biotechnology Research Center, Technical and Vocational Training Organization, Mashhad, Iran

**Keywords:** Quinolinic Acid, Cerebellum, Cognition, Purkinje Cell, Granular Cell

## Abstract

**Objective:**

The cerebellum is a key structure involved in coordinated motor planning, cognition, learning and memory functions. This study presents a permanent model of a toxin produced cerebellar lesion characterized according to contemporary motor and cognitive abnormalities.

**Materials and Methods:**

In this experimental study, slow administration of quinolinic acid (QA, 5 µl of 200 µmol, 1 µl/minute) in the right cerebellar hemisphere (lobule VI) caused noticeable motor and cognitive disturbances along with cellular degeneration in all treated animals. We assessed behavioral and histopathological studies over ten weeks after QA treatment. The data were analyzed with ANOVA and the student’s t test.

**Results:**

The QA treated group showed marked motor learning deficits on the rotating rod test (p=0.0001), locomotor asymmetry on the cylinder test (p=0.0001), dysmetria on the beam balance test (p=0.0001), abnormalities in neuromuscular strength on the hang wire test (p=0.0001), spatial memory deficits in the Morris water maze (MWM, p=0.001) and fear conditioned memory on the passive avoidance test (p=0.01) over a ten-week period compared with the control animals. Histopathological analysis showed loss of Purkinje cells (p=0.001) and granular cell density (p=0.0001) in the lesioned hemisphere of the cerebellum.

**Conclusion:**

Results of the present study show that QA can remove numerous cells which respond to this toxin in hemispheric lobule VI and thus provide a potential model for functional and cell-based studies.

## Introduction

Cerebellar disorders cause motor abnormalities due to infectious illness, injuries or hereditary degenerative processes in the brain. They are characterized by extreme incoordination, gait impairment, disordered eye movements, poor articulation, impaired swallowing, and tremors (1-3).

Beside its function in motor harmonization, the cerebellum acts in cognitive processes such as consideration, verbal studying, working memory, and sensory discrimination. The role of the cerebellum in cognition has generated considerable debate (4- 8). Several studies have examined whether damage in the cerebellum disrupts learning on cognitive tasks similar to that observed in motor learning. For instance, Fiez et al. (9) have proposed that cerebellar damage causes a decline paired-associate learning and semantic recovery (10). It has been proven that, through a link with the thalamus, the cerebellum innervates not only motor regions of the cortex, but also prefrontal and parietal heteromodal association cortices implicated in cognition (11-14). As a result of cerebellar damage, neurocognitive symptoms and a cognitive affective syndrome that consist of slower results and mental disorders have been proven (15). In addition, lesions of the lateral cerebellum impair cognitive functions, resulting in mutism and amnestic aphasia (14). The cerebellar hemisphere in connection with the dorsolateral prefrontal cortex is involved in executive and working memory functions (16-20).

Signs of cerebellar ataxia include significant loss of Purkinje and granular cells. These cells have functional N -Methyl Di Aspartic Acid (NMDA) receptors (21). Purkinje and granular cells involuntarily stimulate or stimulation occurs when cells are infused with glutamatergic afferents (21-23). Quinolinic acid (QA) is a selective NMDA receptor agonist (24). The acute neurotoxic effects of QA in the brain are attached with extracellular Ca^2+^ and direct to hyperphysiological Ca^2+^ condensations into the cell. Enhanced Ca^2+^ levels promote apoptosis pathway with their excitotoxic function (25).

According to previous research, the cerebellum may have a distinct functional topography, with the superior posterior regions mediating certain specific cognitive processes (26, 27). The left superiorposterior lobe of the cerebellum is associated with visual-spatial memory and the right superior-posterior lobe of the cerebellum mediates a planning component of executive functioning (26-28).

As a result of the cerebellar functional topography and anatomical studies of projections between the cerebellum and cerebral cortices it has been hypothesized that the advanced motor tasks would activate regions of the cerebellum to which sensorimotor regions project, namely lobules IV-VI and lobule VIII (29, 30). However, evidences have shown that cognitive tasks predominantly activate lobules VI and VII (31). Therefore, this study sought to develop a permanent model of toxin produced cerebellar abnormalities by focal intra-cerebellar injection of QA into hemispheric lobule VI.

## Materials and Methods

### Quinolinic acid-induced lesions

All experiments were carried out in accordance with the Guidelines of the Animal Care of Ferdowsi University of Mashhad, approved by the University Animal Ethics Committee. Animals were kept on a normal day-night cycle (12 hours/12 hours, lights at 07:00), standard temperature (25 ± 2℃) and humidity conditions. Animals were fed lab chow and tap water ad libitum.

Adult male Wistar rats (270-300 g) were used in this study. Animals (n=20) were anesthetized by a mixture of ketamine hydrochloride (30 mg/kg) and xylazine (4 mg/kg) and then positioned in a stereotaxic apparatus. All rats received a stereotaxic lesion in the right cerebellar hemisphere (lobule VI) via a single 2 μl injection of QA (200 μmol, Merck, Germany) dissolved in 0.1 M phosphate buffer saline (PBS) which was administered by a 5G-Hamilton syringe at the following coordinates: AP=11.96 mm, ML=2.96 mm, and DV=4.0 mm from the bregma. QA was slowly injected over a period of 5 minutes; the needle was left in position for another 4 minutes and then gently removed. Rats (n=20) in the vehicletreated group received injections of PBS in the same stereotaxic coordinates. After recovery from surgery, rats were returned to their cages and assessed daily for neurological function.

### Motor function assessment

#### Rotarod

The accelerating rotarod apparatus (47700, Ugo Basile, Italy) was used to assess balance and coordination by measuring the amount of time the animal was able to remain on a longitudinally rotating rod. During the training period, rats were placed on the rotarod starting at a speed 5 rpm which was slowly increased to 40 rpm. The maximum observation time was 5 minutes. Animals were tested with four trials per week with a 1 hour between trials. The latency to fall during the observation period was recorded. Rotarod data was analyzed by ANOVA statistical tests (n=20).

#### Cylinder test

One week after QA injection, all rats were assessed for motor function via the cylinder test. The cylinder encourages use of the forelimbs for vertical exploration and landing after a rearing movement (32, 33). Animals were placed in a transparent plastic cylinder (20 cm diameter, 30 cm height) and videotaped until they had performed 20 rears while making contact with the walls of the cylinder. The test was carried out in two successive trials with one hour interval over a ten-week period (one testing session per week) following the QA injection. The proportion of paw use (impaired/normal) was counted (total of 20 paw placements per trial).

#### Beam walking test

The beam walking task consisted of placing the rats on an elevated (100 cm) narrow wooden beam (3 cm wide) held 30 cm above the ground with a large 40 cm^2^ platform at one end (34). Animals were trained to traverse the beam prior to testing. An animal was considered to have mastered the task when it spontaneously crossed the entire 100 cm length of the beam without falling off, for three consecutive trials each day over a three-day period. The distance moved along the beam was recorded as 100 cm if the animal traversed the entire beam successfully or was measured from the rear of the hind limbs at the point where the animal fell. The total time taken to cross the beam, including the time where animal was immobile on the beam, was recorded along with the time spent moving. Testing was conducted for three trials per day, for a 10-week period.

#### Hanging wire test

This task was used as a measure of muscular strength and motor neuron integrity (35). Rats utilized their forelimbs to suspend their body weight on a wire (0.5 cm in diameter, 60 cm length) that was stretched between two posts and located 20 cm above a foam pillow. The time (in seconds) until the rat fell was recorded. A score of zero was assigned if the rat fell immediately and 60 seconds was the timeout period for a 10-week period after QA injection.

### Cognitive function tests

#### Morris water maze: Acquisition of spatial learning

Spatial learning was assessed in a Morris water maze (MWM) apparatus. This test was shown to be a measure of cognitive function and dysfunction after QA injection (36, 37). The maze consisted of a plastic pool (180 cm diameter, 60 cm height) filled with tap water (26 ± 1˚C) to a depth of 28 cm that was located in a room with salient visual cues. The hidden platform was a clear Plexiglas stand positioned in the southeast quadrant and held constant for each rat. Animals were tested with four trials per day, over five consecutive days at three weeks post injection. Each trial was initiated by placing the animal at one of four randomly chosen locations, facing the wall of the tank. Animals were allowed to search for the hidden platform for a period of 60 seconds. If an animal failed to find the platform, it was placed on the platform by the researcher and allowed to remain there for a period of 30 seconds before being returned to a warming cage for a five-minute period between trials. For each trial, movement within the maze was monitored by a video camera linked to tracking software. The latency to platform was calculated as the time necessary to locate the hidden platform. Using the tracking software, we calculated time spent swimming in circles and swim speed. Animals’ performances in spatial acquisition were analyzed using the repeated measure one-way ANOVA.

#### Passive avoidance test

We used the passive avoidance learning test based on negative reinforcement to examine long-term memory (38). The passive avoidance test was conducted by using an apparatus that consisted of two separate chambers (light and dark chambers) connected to a computer. Each chamber was separated by a small guillotine door and grids were attached on the floor in the dark chamber. This test comprised the following three sessions at five weeks post-injection. First, a step through ‘Trials to Criterion’ procedure was used in the training session on the first and second day. During the test session (day three), rats were individually placed in the light chamber. Immediately after the rats entered the dark chamber, the door was locked and an electrical stimulation of 2 mA and 50 Hz was applied for 2 seconds. In the retention session (day four) which was performed 24 hours later, rats were again placed in the light chamber and the time spent in this chamber before entering the dark chamber, within five minutes, was measured. The latency in the retention session was expressed graphically and used in data analysis.

#### Animal sacrifice and tissue analysis

After completion of behavioral testing (at the end of ten weeks post-injection), the animals were terminally anesthetized with an intra-peritoneal injection of a mixture of ketamine hydrochloride (100 mg/kg) and xylazine (5 mg/kg) and intracardially perfused with 0.9% saline followed by 10% phosphate-buffered formalin. After fixation, the brains were removed and post-fixed in 10% phosphate-buffered formalin for seven days. The brains were then blocked to remove the forebrain, after which the cerebellum was paraffin-embedded and sectioned on a microtome in 5 μm sections. Sections were deparaffinized, serially hydrated, stained with hematoxylin-eosin (H&E), and then cover-slipped for tissue analysis.

### Neurotoxic lesion area

Using a macro objective (Nikon 1X, Nikon, Japan), a photomicrograph (Nikon Eclipse E600) was obtained of each coronal section throughout the cerebellum of each animal by a systematic random procedure. Image analysis software (ImageJ, National Institute of Health) was used to outline the lesion, if present, and to calculate the area for each section. Using Cavalieri’s principle an estimate of total neurotoxic lesion areas per animal was calculated in cubic millimeters.

### Purkinje cell counting

Design-based stereology was performed to count Purkinje cells in lobule VI in H&E stained sections. A total of 15 sections were used for obtaining Purkinje cell counts. The Purkinje cell layer was identified at ×2 magnification on live microscopic images displayed in a stereological computer microscopy system. Purkinje cells with a visible nucleolus were counted by visual inspection under a ×40 objective, Purkinje cells were marked if they were positive. We counted the total number of Purkinje cells in all sections. For each animal, we randomly chose and analyzed 15 sections that contained all lobules of the cerebella hemispheres (intact and lesion hemisphere). All Purkinje cell numbers were averaged to obtain one value per lesion and intact hemispheres of groups.

### Granular cell density

We obtained estimates of the total numbers of H&E stained cells in the granular cell layer of the cerebellum in the both hemispheres per rat. Light microscope photographs were taken of the granular cell nucleus. The images were processed with Image J software. In each cerebellar section, neurons were counted at ×100 amplification. The size of the counting frames was 50×50 mm for grids of 120×120 mm. Neurons were counted if their somata were found within the counting frames (three frames were created by the software and placed at the intersections of a grid) or overlapping its top-right border. We obtained an unbiased estimate of the granular cell number for each section. H&E staining was performed on 9-10 sections sampled in an even distribution across the tissue, which provided appropriate sampling to apply the stereological cell count method (39).

### Statistical analysis

The data were collected by observers blinded to treatment conditions and analyzed using repeatedmeasures ANOVA, with time as the repeated measure. All group comparisons were analyzed using either the student’s t test or Tukey’s post hoc test to account for multiple comparisons and to determine which groups significantly differed from one another. A significance level of p<0.05 was used for all tests. Values are presented as mean ± SEM.

## Results

### Motor performance

#### Beam walking test

**Fig 1:**
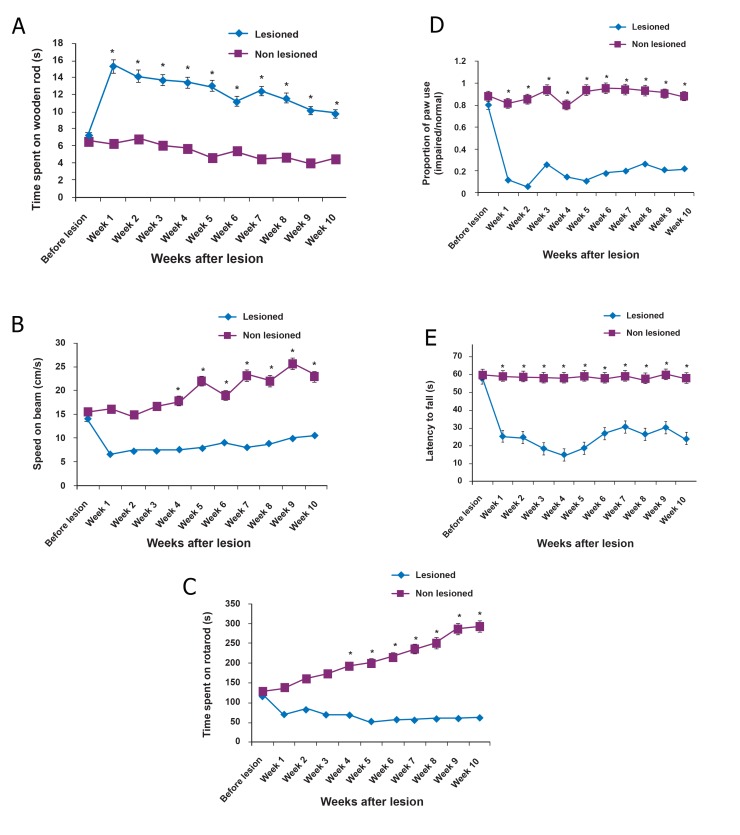
Motor performance: By ten weeks post-lesion, the quinolinic acid (QA)-lesioned group began to develop weakness on the beam walking test, as evidenced by the time spent on the beam (A) and the speed (B) when compared with nonlesioned rats (vehicle treated rats, *p≤0.0001). In the motor learning task, rotarod (C), the QA-lesioned group showed a behavioral deficit from the first week which continued up to ten weeks (*p≤0.0001). Complete loss of motor coordination was observed in the QA-lesioned group in the rotarod test. The lesioned group failed to stay on the rotating rod even after ten weeks. Animals’ function on the cylinder test (D) after QA lesioning were assessed as the proportion of limb use (impaired/normal). After a QA lesion in the right cerebellum’s hemisphere, rats in the lesioned group rarely used their impaired forelimb to explore the cylinder wall during the ten weeks of testing compared with vehicle-treated rats (*p≤0.0001). In the hanging wire test (E) QA-treated rats showed less latency to fall from the wire than non-lesioned rats (vehicle treated rats,* p ≤ 0.0001). All error bars indicate standard error of mean (SEM).

There were statistically significant differences in the based beam walking test between groups. Longer beam-walk times were observed in the lesioned rats (n=20, Fig 1A) compared to the controls (Fig 1B) for all times tested (n=20, p≤0.0001 vs. non-lesioned for all motor tests).

#### Rotarod test

All animals (n=20) showed deterioration in the rotarod scores at one week post-injury. Rotarod motor testing revealed significant decreases in falling latency in QA-injected rats compared with vehicle treated rats (n=20) during ten weeks after the lesion (Fig 1C, p≤0.0001).

#### Cylinder test

Vehicle-treated rats (n=20) made a majority of double contacts on the cylinder wall during their explorative behavior and used each limb to explore the cylinder with the same frequency and proportion of limb use (impaired/normal) was 0.8966 ± 0.011. After infliction of the QA lesion, lesioned rats (n=20) held their impaired forelimb in a rigid tight position and rarely used the impaired forelimb to explore the cylinder. Therefore, the lesioned group showed a dramatic decrease in the proportion of double contacts due to decreased spontaneous use of the contralateral forepaw (Fig 1D, p≤0.0001).

#### Wire hanging test

The wire hanging ability of the rats with QA lesions decreased. The mean latency time to fall of the QA-injected group (n=20) was significantly less than that of the vehicle treated rats (n=20) during ten weeks post-lesion (Fig 1E, p≤0.0001). These results indicated that the QA injection significantly disturbed neuromuscular strength in this model.

### Cognitive performance

#### Morris water maze

The data for this test of spatial memory showed statistically significant differences in the latency to reach the fixed invisible platform by the MWM test (F value: 128.987, p≤0.001). For both groups, there were comparable latency and path length measures from day one in learning to locate the platform.

**Fig 2:**
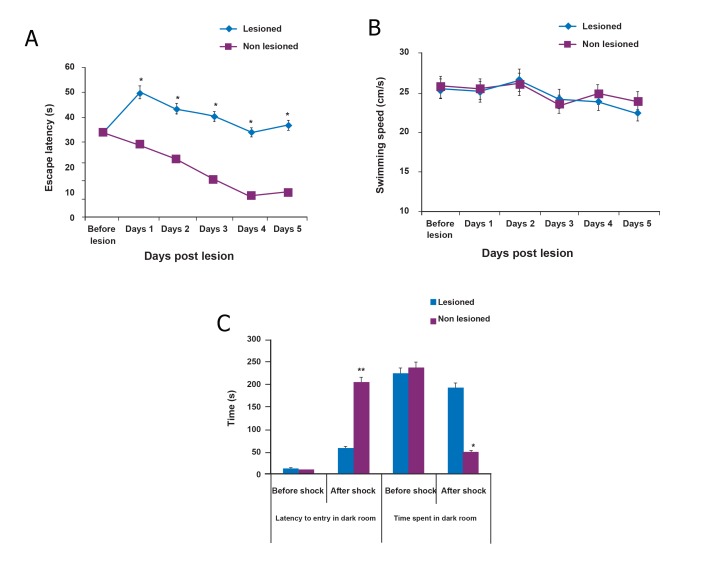
Cognitive performance: Three weeks post-lesion, animals were tested in the Morris water maze (MWM) task. Animals were tested using four trials per day for latency (A) and speed (B) to reach the hidden platform, which was recorded after each trial. Quinolinic acid (QA)-treated rats showed longer latencies to the hidden platform than non-lesioned rats (vehicle treated, F value: 128.987, *p≤0.001). Swimming speed was not significant between the groups (F value: 1.448, p≤0.233). A comparison of the delay times (to enter) and total stay times in the dark electrical foot shock box (before and after shock) was tested in the passive avoidance test (C). These profiles showed statistically significant differences between vehicle and QA-treated groups at 48 hours after shock (**p=0.008 for latency to entry and *p=0.016 for time spent in dark room). There were no significant differences before and after the shock in each group.

Post-hoc testing demonstrated significantly increased latencies to locate the hidden platform in the QA-injected group compared to the control group on subsequent days (Fig 2A). Swim speed did not significantly differ between the groups (F value: 1.448, p≤0.233, Fig 2B) which suggested that accurate assessment of place learning was not precluded by extraneous factors such as motor disparities. These data suggested that QA-injected animals (n=20) had more difficulty in learning the location of the platform compared with control animals (n=20) and exhibited a memory deficit after the hemispheric lobule VI injury.

#### Passive avoidance test

We determined the putative differences in the passive avoidance test for the two experimental groups by measuring the time taken by the rat to enter the dark compartment after the door was opened. This test was conducted at five weeks post-lesion (Fig 2C). Based on our data, there were no significant differences between groups in the time spent before entering the dark compartment and time in the dark compartment in the acquisition session. The latencies were 12.11 ± 0.21 seconds (n =20) for the vehicle-treated group (control) and 14.67 ± 0.3 seconds (n=20) for lesioned animals. The total staying time in the dark electrical foot shock box was 237.60 ± 10.4 seconds for the control group and 224.84 ± 9.08 seconds for the QA-injected group. In contrast, for the retention session performed 48 hours after the first session, two-way ANOVA showed a significantly longer retention time for non-lesioned rats that moved into the dark compartment compared with the lesioned group (F value: 8.795, p=0.008). Control group showed longer latency (204.87 ± 11.28 seconds) whereas the QA-injected group showed a significantly lower value (58.604 ± 4.003 seconds). These results suggested a difference in the retention performance of the control versus lesioned group. The QA-injected group remained the longest (193.5 ± 9.31 seconds) in the dark box during the entire follow-up period; the control rats stayed 50.29 ± 4.3 seconds (F value: 6.931, p=0.016, Fig 2C). Therefore, it could be concluded that in the passive avoidance test, QAinjected animals had significant memory deficits compared to the control animals.

**Fig 3:**
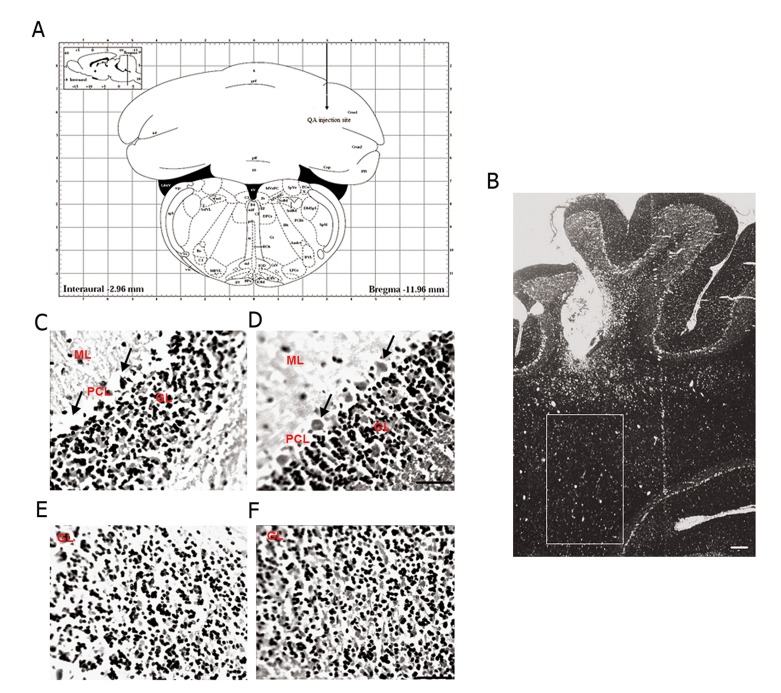
Hematoxylin-eosin (H&E) staining: Quinolinic acid (QA) injection site in schematic (A) and real (B) pictures. In the lesioned hemisphere there is Purkinje cell loss (C) compared to the right cerebellar hemisphere of control rats (D). Granular cell density is decreased in the right cerebellar hemisphere of QA-treated rat (E), while it is intact in right hemisphere of vehicle-treated rat (F). The scale bar is 100 μm. Arrows shows PCL. ML; Molecular layer, PCL; Purkinje cell layer and GL; Granular layer.

### Histological analysis

#### Purkinje cells counts and neurotoxic lesion area

**Fig 4:**
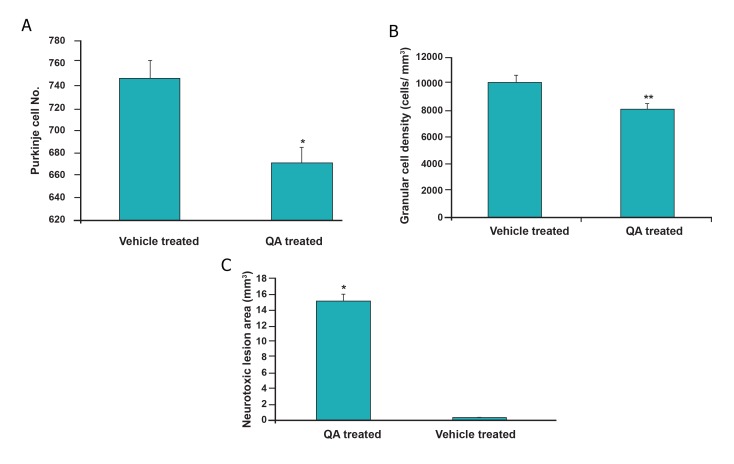
Stereological analysis: Average of Purkinje cell numbers in 15 random sections of the right hemisphere in the quinolinic acid (QA) and vehicle- treated groups after hematoxylin-eosin (H&E) staining (A). Histograms showed that the number of Purkinje cells was significantly reduced in right hemisphere of QA-treated rats compared with vehicle treated rats as controls (F value: 11.738, *p≤ 0.001). Granular cell density was decreased in right hemisphere of QA-treated rats compared with controls (B, F value=45.785, **p≤0.0001). In the neurotoxic lesion area (C), a significant between-group difference was found (F value: 15.816, *p≤0.001).

H&E staining demonstrated that the numbers of Purkinje cells in the right hemispheres of animals in the QA-injected group were less compared to the number of Purkinje cells in right hemisphere of vehicle treated rats (Fig 3C). After stereological analysis, we observed a significant decrease in the total number of Purkinje cells in the QA-injected group compared to control animals (Fig 4A, F value: 11.738; p≤0.001). We evaluated the neurotoxic lesion area and noted a significant difference between the groups (Fig 4C, F value: 15.816; p<0.001). As seen in figure 3B, compared to vehicle treated rats, animals that received a single dose of QA (5 μl of 200 μmol) in the right cerebellar hemisphere demonstrated significantly increased mean neurotoxic lesion areas (15.141 ± 2.1 mm^3^) compared to the control group (0.232 ± 0.002 mm^3^).

#### Granular cell density

Based on histological analysis, we observed decreased granular cell density in the QA-treated group compared with control rats (Fig 3E, F). Among animals at ten weeks post-injury, significant group differences existed in terms of the granular cell layer estimated cell density (Fig 4B, F value=45.785, p≤0.0001).

## Discussion

Most neurodegenerative diseases, including cerebellar degeneration, lead to severe behavioral abnormalities in mammals. Although classically considered to be involved only in motor coordination, the cerebellum has more recently been implicated in cognitive control (40, 41). The right cerebellum in relation to the dorsolateral prefrontal cortex is involved in executive and working memory operations such as subvocal rehearsal mechanisms of verbal working memory (40-42). Neuropsychological studies in patients with degenerative cerebellar ataxia have shown the presence of cognitive dysfunction, mainly of the executive type (43). Our results have indicated that the QA lesioned cerebellar hemisphere contributed to some of the cognitive deficits observed in the MWM task and passive avoidance test. Further studies are necessary to determine all of the cortical areas of the cerebellum that participate in non-motor functions.

Stoodley and Schmahmann (31) have proposed a separation among sensorimotor and cognitive cerebellum. Sensorimotor cerebellum includes the hemispheres of the frontal lobe, lobulus simplex (lobules HV and HVI), and paramedian lobule (HVIII). The cognitive cerebellum consists of HVI, Crus I and II and the lobule HVIIB of the posterior lobe. In a meta-analysis of neuroimaging studies of functional topography within the cerebellum, both lobule VI and crus I of VIIA in the superior-posterior lobes have been found to be consistently activated during executive function tasks (30). Hemispheric lobule VI was specifically activated during spatial tasks (29, 44, 45). We confirmed that hemispheric lobule VI was implicated in memory processing, skill learning and motor coordination in the rat model of a hemispheric lobule VI lesion.

However, recent reports have shown that Purkinje cells in folia VI-IX are more vulnerable because of their motor coordination function and that the less vulnerable folia are involved in cognition (46). In the current study’s model, QA could destroy a large area of hemispheric lobule VI (Fig 3B), causing a decrease in Purkinje and granular cells. QA specifically affected memory task as well as motor coordination.

The beam walking test, rotarod, and hanging wire test have been used in order to differentiate between animals that have lost different cell populations in the cerebellum. These tests show sensitivity to cerebellar lesions and different levels of severity have been delineated depending on the type of cerebellar degeneration studied (47, 48).

The cerebellar lesions in QA-injected rats showed time-related deficits. We examined motor deficits by rotarod, beam walking, hanging wire and cylinder tests at ten weeks post-injection. The results showed the presence of permanent motor impairments and functional deficits in the QA-treated group even at ten weeks after injury.

QA is a potential model for cerebellar disorder. The NMDA receptor agonist causes partial loss of Purkinje cells that mimic progressive clinical deterioration. We have recently shown that this model is suitable for stem cell transplantation (49). This is a significant advantage over genetic models of disease where there is a total loss of Purkinje cells together with a variable number of surviving supporting cells (50).

## Conclusion

Our data indicate that local lesions in cerebellar lobule VI lead to impairments in motor performance and memory functions. The effect of QA on hemispheric lobule IV has enabled us to develop an acute focal neurotoxic lesion in the cerebellum. Future anatomical, physiological and imaging studies in animal models and humans will clarify the precise role of cerebellar organization and its connections in motor learning, sensorimotor control and cognition tasks. This paper has attempted to show some aspects of cerebellar functions in hemispheric lobule VI and provide further support for a cerebellar role in both motor and cognitive tasks, in addition to a better establishment of the existence of lobule VI function in the cerebellum.
